# Case report: Depressive disorder with peripartum onset camouflages suspected intracranial tuberculoma

**DOI:** 10.3389/fpsyt.2022.932635

**Published:** 2022-09-29

**Authors:** Halwa Zakia, Shelly Iskandar

**Affiliations:** ^1^Faculty of Medicine, Padjadjaran University, Bandung, Indonesia; ^2^Department of Psychiatry, Faculty of Medicine, Padjadjaran University, Bandung, Indonesia

**Keywords:** depression, intracranial tuberculoma, meningitis tuberculosis, neuroimaging, postpartum depression

## Abstract

**Background:**

Co-occuring psychological symptoms and rare medical illnesses like intracranial tuberculoma add difficulties in diagnosing and treatment. The present report discussed a rare case of a young woman with tuberculoma and depressive disorder with peripartum onset.

**Case presentation:**

The patient was a 22-year-old woman. She underwent a cesarean section due to premature rupture of the membrane. She had developed behavioral changes for 3 months before admission and had been brought to general practitioner for several times during pregnancy. Her symptoms worsen after she underwent a cesarean section. She refused to answer doctor's and family's questions and she showed symptoms of depression. She was diagnosed with depressive disorder with peripartum onset and treated according to her diagnosis. However, she also experienced intermittent headaches which lead to suspicion that there was an organic cause for her complaints. Computed tomography (CT) was done and revealed suspected intracranial tuberculoma and meningitis. She died due to respiratory distress before she got adequate treatment for her illnesses.

**Conclusion:**

The possibility of neurocognitive disorder due to infection such as intracranial tuberculoma should be considered as an additional diagnosis (co-occuring disease) or differential diagnosis in people who develop psychiatric symptoms, especially in areas with high tuberculosis incidence.

## Introduction

Central nervous system (CNS) tuberculosis (TB) is uncommon, but is one of the most dangerous types of human mycobacterial infection (1). In developing countries, intracranial tuberculoma accounts for 5%−30% of all intracranial lesions (2). Intracranial tuberculomas manifest clinically in a variety of ways, depending on the location, size, and number of lesions (3). CNS TB has a worse prognosis than pulmonary TB when therapy is delayed (4). In the case of CNS TB, neuroimaging aids diagnosis, risk assessment, and clinical course monitoring (5). Prenatal depression, “baby blues,” and postpartum depression are all forms of depressive disorder with peripartum onset that range in intensity and onset time. It affects 10%−20% of the population (6) and can occur at any time throughout pregnancy, notably in the third trimester, or several weeks to months after childbirth. Around 10% of pregnant women and 13% of postpartum women worldwide suffer from a mental illness, with depression as the most common form (7). Depressive disorder with peripartum onset is defined as depression which onset of mood symptoms occurs during pregnancy or in the 4 weeks following delivery (8). To rule out an organic cause, laboratory tests and a complete physical examination should be performed (9). Rare medical illnesses like intracranial tuberculoma can sometimes be mistaken for postpartum psychological problems (10) or can exist as co-occurring disease.

The present report discussed a rare case of a young woman with suspected tuberculoma who developed depressive disorder with peripartum onset. This work has been reported in line with case report (CARE) guidelines (11). We believe this work can support healthcare providers to consider neurocognitive disorder as differential diagnosis or additional diagnosis/ co-morbidity when facing patients with psychiatric diagnosis.

## Case description

**Figure 1:**
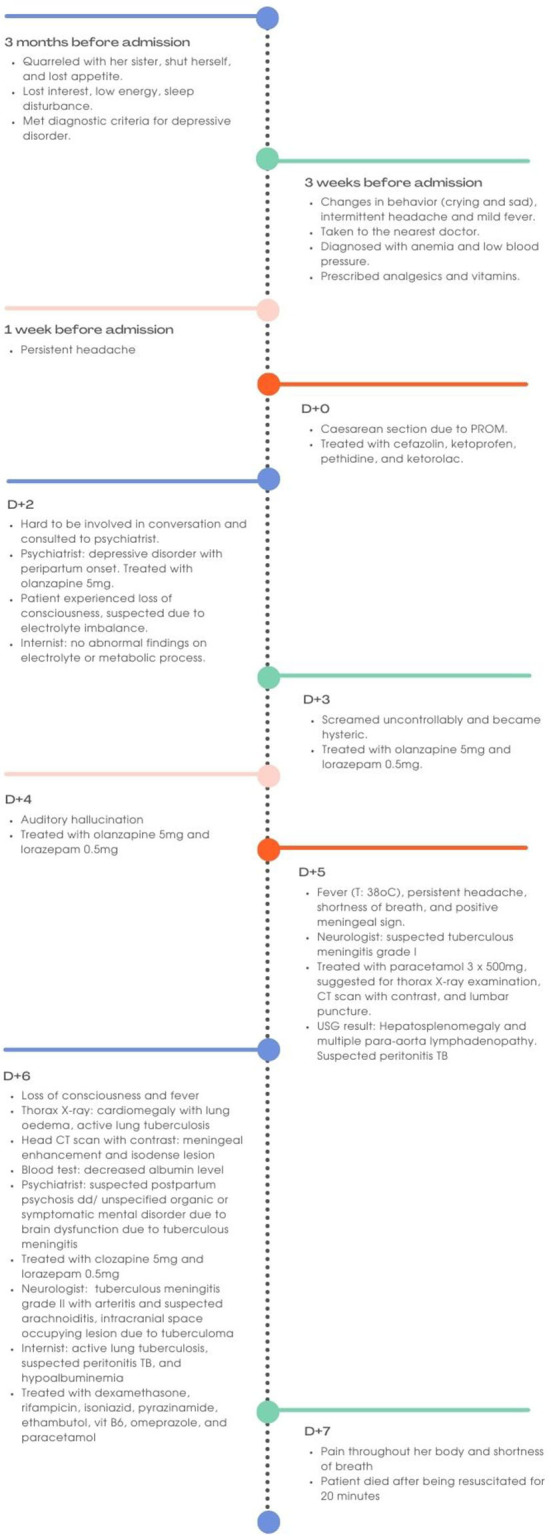
Timeline for the course of the case.

A 22-year-old Asian woman developed marked changes in behavior and personality following the delivery of her first baby (Figure 1). She gave birth in the 33rd−34th week of gestation due to premature rupture of the membrane (PROM). The baby weighted 2,000 g with body length of 43 cm and APGAR score of 6 and 8 for 1 and 5 min, respectively.

The patient ignored the doctor's and family's questions after the delivery. She was known to be irritable due to the emergency cesarean section. She felt sleepy; however, she could not fall asleep. Her breastmilk production was very low which made her even more irritated. She also looked confused and kept babblings meaninglessly to herself. She admitted that she heard the ghost's voices which could not be heard by others.

Three months before admission, her sister posted her pregnant belly on social media without her permission, which induced conflict between the two. She shut herself for 2 months in her room and refused to eat, therefore she lost some weight. Three weeks before admission, the patient sometimes was seen crying without any specific reasons. She felt sad, fatigued, and lethargic. She also had a conflict with her husband because he ignored her request on getting health insurance. Set aside, she also got mad at her mother for no apparent reason. In addition, she experienced frequent intermittent headaches associated with mild fever which had been felt for 3 weeks before admission. The patient had been brought to the nearest health care facility for several times to check her complaint of headache. The doctor found that she experienced anemia and low blood pressure which is common in a pregnant woman. The patient then was prescribed analgesics and vitamins. There was no history of mental illness in the patient and her family. There was no known head trauma. Patient's family believe those symptoms related to her pregnancy.

At the time of admission, after having a cesarean section due to PROM, she had fever (temperature: 38.7°C) with other physical examinations showed no abnormal findings. Patient was treated with cefazolin, ketoprofen, pethidine, and ketorolac regarding to her condition. Two days after admission, patient experienced loss of consciousness which was suspected due to electrolyte imbalance by physician in Emergency Department at the time of admission. Thus, she was followed up by internist. However, there was no abnormality in either electrolyte or metabolic process.

Biochemical investigations were done several times since the patient's admission. After patient lost her consciousness, 2 days after admission, the hemoglobin level was 8.2 g/dl (reference range: 12.3–15.3 g/dl), platelet count was 308 × 10^9^/L (reference range: 150–400 × 10^9^/L), and white blood cells count was 11 × 10^9^/L (reference range: 4–11 × 10^9^/L). Other laboratory results including calcium, glucose, blood urea nitrogen, and creatinine were normal.

She then turned into hypoactive with a negative attitude. The rapport was inadequate. She remained silent while being asked by physicians. Due to her behavioral changes, she was consulted to Psychiatric Department. According to her complaint, she was diagnosed with depressive disorder with peripartum onset. Depressive disorder was diagnosed because the patient had depressed mood most of the day; diminished interest or pleasure in all, or almost all, activities most of the day; significant weight loss; fatigue or loss of energy; feelings of worthlessness in the last 3 months. The patient was treated with olanzapine 5 mg per day. The next day, the patient screamed uncontrollably and became hysteric. Due to her condition, lorazepam 0.5 mg per day was added into her therapy regimen. Later, she developed auditory hallucination of hearing ghost's voices which no one else can hear.

Five days after admission, she was then consulted to a neurologist for further assessment of her headache. From the assessment of the neurologist, the Glasgow Coma Scale (GCS) was 14 with a positive meningeal sign. Cranial nerve (CN) examinations revealed paresthesia on the right side of her face. Motoric examination revealed tetra paresthesia with positive pathological reflexes. She was suspected with tuberculous meningitis grade I and suggested to undergo thorax X-ray examination, computed tomography (CT) scan with and without contrast, and lumbar puncture.

**Figure 2:**
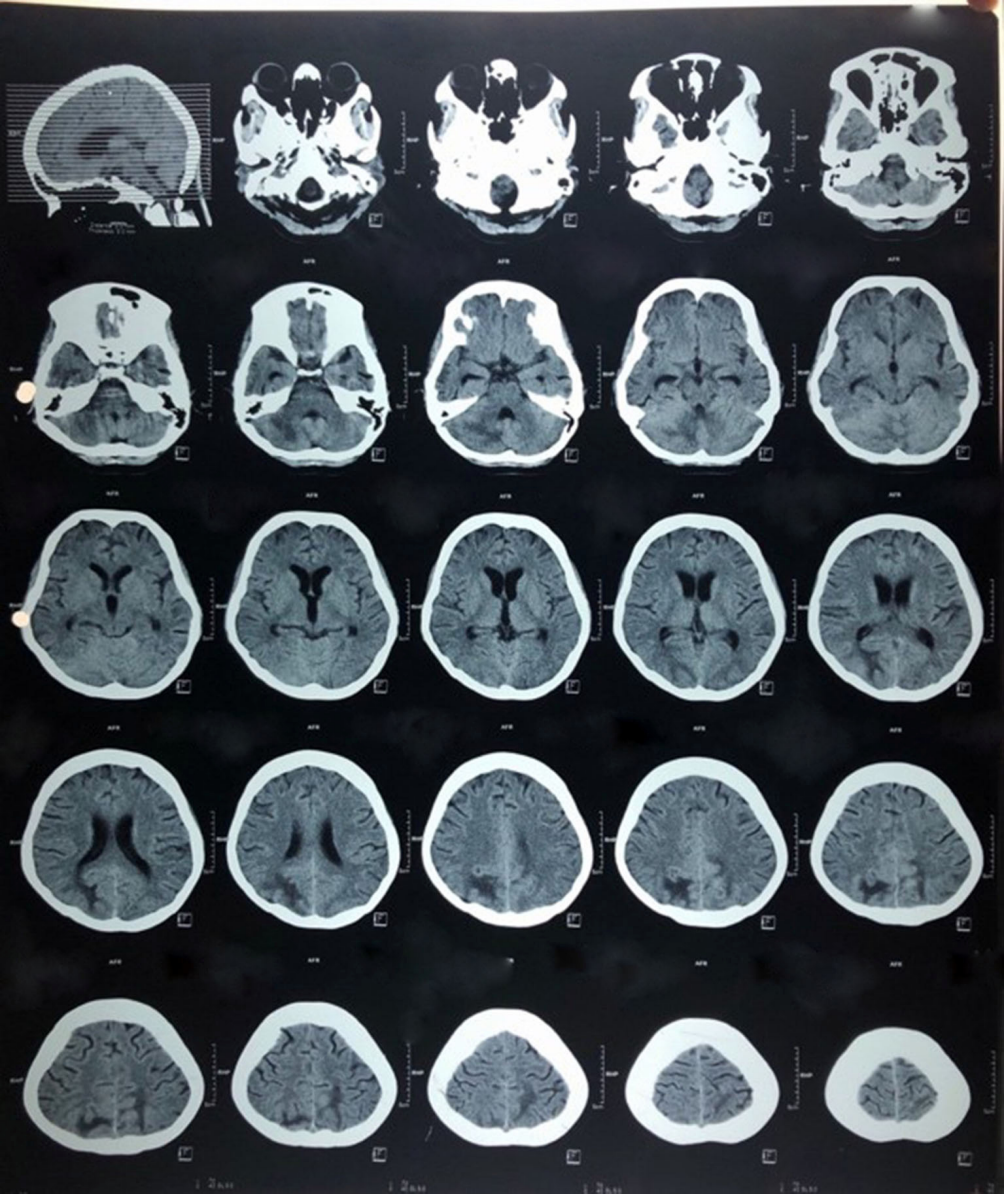
Axial non-enhanced computed tomography scan showing multiple lesions at subcortical right and left occipital lobe with meningeal enhancement.

The following day, a non-enhanced CT scan of her head revealed hypodense lesions at right subcortical occipital lobe, left parietooccipital lobe, and right cerebellum accompanied by hydrocephalus. Those lesions could be an infarct due to embolization with differential diagnosis of posterior reversible encephalopathy syndrome (PRES) and tuberculoma. In an enhanced CT, we found an overlie meningeal enhancement at sulci cortical right occipital lobe, bilateral sulcus cortical temporal lobes, tentorium cerebelli, ambient cisternae, basalis cisternae, bilateral Sylvia fissures, anterior and posterior interhemispheric fissure with hydrocephalus communicans, which suggested an additional diagnosis of meningitis (Figure 2). She also underwent several other imaging to support the diagnosis of possible underlying disease. An abdominal ultrasound scan revealed hepatosplenomegaly and multiple paraaortic lymphadenopathies, which added additional suspected diagnosis of tuberculous peritonitis. A chest X-ray was performed and suggested active pulmonary tuberculosis and cardiomegaly with lung oedema.

Based on the latest result that day, psychiatric diagnosis was revised to peripartum depression and neurocognitive disorders due to brain dysfunction due to tuberculous meningitis and patient was treated with clozapine 5 mg and lorazepam 0.5 mg. Also, neurologist updated the diagnosis into to tuberculous meningitis grade II with arteritis and suspected arachnoiditis, intracranial space occupying lesion due to tuberculoma. Patient was soon treated with rifampicin, isoniazid, pyrazinamide, ethambutol, dexamethasone, vit B6, omeprazole, and paracetamol.

Seven days post cesarean section, the patient suddenly complained of pain throughout her body. She developed shortness of breath with a respiratory rate up to 28 x/m, fever (temperature: 38.9°C), low blood pressure 90/70 mmHg, and decreased oxygen saturation (SpO_2_ 80%). The patient then stopped breathing. Twenty minutes after being resuscitated, the patient did not show any response and was declared dead with suspected pulmonary embolism as cause of death. However, confirmation was not possible because a thorough postmortem study was not performed.

Lumbar puncture was scheduled to be done on the patient after an enhanced CT scan result was obtained. However, patient died before the test was held. We also did the acid-fast bacilli (AFB) test; however, the patient passed away before the result came out.

## Discussion

Tuberculoma can appear as a single or several lesions with sizes varying from less than a centimeter to several centimeters (12). In a review of 102 cases, pregnancy, puerperium, and diabetes mellitus were identified as risk factors for intracranial tuberculoma (13). Immune changes during pregnancy can result in weakened cell-mediated immunity and increased susceptibility to diseases like TB (14). Some studies revealed that the immunosuppression related to pregnancy could lead to infectious processes similar to that occurs in immunocompromised hosts (15, 16). The T-helper 1 (Th1) proinflammatory response is suppressed during pregnancy, increasing susceptibility to new infections and reactivation of TB, but after delivery, Th1 suppression reverses, exacerbating symptoms and making women twice as likely to develop TB in the early postpartum period compared to women who had not been pregnant (17, 18).

In addition of immunosuppression state of our patient due to pregnancy, she also had experienced depression 3 months before the signs and symptoms of tuberculoma appeared. Depression is well-known as one of the factors that contribute to reduced immunity. Natural killer (NK), neutrophil, and lymphocyte activity are reduced in depression, and stressful situation (19). This condition led patient to be more vulnerable to acquire new infection or to have reactivation of the existing infection, as in our patient.

Multiple inflammatory molecules have been found to be expressed differently in depressed patients' peripheral blood and/or CSF. For instance, the serum of depressed patients has higher levels of pro-inflammatory cytokines and chemokines like interleukin (IL)-1, IL-2, IL-6, IL-12, tumor necrosis factor (TNF), interferon (IFN), CXC chemokine ligand 4 (CXCL4), and CXCL7, as well as other inflammatory reactants like monocyte chemoattractant protein (MCP)-1 and C-re 14–20,23. In contrast, depressed people have lower blood levels of anti-inflammatory cytokines and chemokines such IL-18, IL-4, Transforming Growth Factor beta 1 (TGF-1) and C-C motif ligand 4 (CCL4) (20).

Our patient was first diagnosed with depressive disorder with peripartum onset based on DSM 5 criteria. The patient experience depressed mood, sleep disturbance, and lack of energy, which onset starts 3 months before admission. She also experienced diminished interest in almost all activities nearly every day. She had difficulty on falling asleep and felt restless. There were also several psychosocial problems before the onset of the disease which strengthen the possibility of depressive disorder with peripartum onset. Misdiagnosis in the case of tuberculoma often occurs since intracranial TB is infrequent and sometimes accompanied by non-specific symptoms in pregnancy period that make diagnosis challenging (10, 21). The condition of intracranial TB can be subacute or chronic, with a duration ranging from weeks to months. Headache (intracranial hypertension), seizures, impaired mental state, and papilledema are some of the most prevalent signs and symptoms of tuberculoma in adulthood (22).

Proper anamnesis to exclude all possible differential diagnosis and mental status examination included inspection in level of consciousness, appearance and general behavior, speech and motor activity, affect and mood, thought and perception, attitude and insight, and cognitive ability should be performed carefully (22). A change in personality and behavior such as refuse to talk and negative attitude toward examiner in this patient can be a hallmark of frontal lobe disease, but it can also happen when other parts of the brain are damaged (23). Therefore, to assess the abnormality in the brain, modality of imaging should be performed.

The CT scan and/or MRI remain the gold standard for diagnosing CNS TB. The presence of calcification with ring enhancement is called the “target sign,” which is specific for tuberculoma (24). Multiple lobulated or aggregated ring appearances can help differentiate tuberculomas from brain tumors (25). However, a tumor, a fungal granuloma, or an abscess may not always be distinguished from tuberculoma.

Accompanying meningitis was found in our case. Meningitis and tuberculoma have a reciprocal causation relationship. Tuberculoma may occur when CSF infection spreads to the nearby parenchyma through cortical veins or Virchow-Robin spaces (26). Meningitis can occur when a tuberculoma breaks into the subarachnoid space (27). We also hypothesized that this patient's fever was mostly related to the presence of meningitis. Another possible source of the fever is puerperal infection which could be experienced by the patient.

Our patient experienced loss of consciousness. Tuberculoma evolves over 1–2 weeks. Thick gelatinous exudate infiltrates the cortical and meningeal blood vessels, resulting in inflammation, obstruction, or infarction. This may lead to hydrocephalus and in severe case can lead to a coma (28).

The patient's death was suspected due to pulmonary embolism. It was known that cesarean section was one of the risk factors for the occurrence of pulmonary embolism in woman during postpartum period (29). The rate of postpartum pulmonary embolism without an antecedent thrombotic event was 0.45 per 1,000 births (29). A study of hemostatic changes in the puerperium and a case control study reported 95% of postpartum pulmonary embolism occurred within the first 4 weeks (30, 31). Thromboembolic disease in pregnancy occurred due to venous stasis, vascular injury, and hypercoagulable state (Virchow triad) (32).

The treatment of intracranial tuberculomas is similar to that of tuberculous meningitis or miliary TB and includes TB therapy and corticosteroids (33). The World Health Organization (WHO), Centers for Disease Control and Prevention of America (CDC) and the British Thoracic Society (BTS) recommend a 9–12 months course of TB treatment for CNS TB (33). However, we did not have the opportunity to provide TB treatment and corticosteroids to our patient before she died.

## Conclusion

Screening for neurocognitive disorder due to infection such as intracranial tuberculoma should be considered as differential diagnosis or additional diagnosis (co-morbidity) of psychiatric symptoms, especially in areas with a high tuberculosis incidence. Intracranial tuberculoma can sometimes be mistaken as postpartum depression. The delay in diagnosis could severely worsen the prognosis.

## Data availability statement

The raw data supporting the conclusions of this article will be made available by the authors, without undue reservation.

## Ethics statement

The studies involving human participants were reviewed and approved by Padjadjaran University. The patients/participants provided their written informed consent to participate in this study. Written informed consent was obtained from the patient's legal guardian for the publication of any potentially identifiable images or data included in this article.

## Author contributions

All authors listed have made a substantial, direct, and intellectual contribution to the work and approved it for publication.

## Funding

The authors disclosed receipt of an Internal Grant of Universitas Padjadjaran for financial support of publication of this article.

## Conflict of interest

The authors declare that the research was conducted in the absence of any commercial or financial relationships that could be construed as a potential conflict of interest.

## Publisher's note

All claims expressed in this article are solely those of the authors and do not necessarily represent those of their affiliated organizations, or those of the publisher, the editors and the reviewers. Any product that may be evaluated in this article, or claim that may be made by its manufacturer, is not guaranteed or endorsed by the publisher.
